# Supplementary material for: Associations between self-reported and objective face recognition abilities are only evident in above- and below-average recognisers
Source: PeerJ. 2021 Jan 11;9:e10629. doi: 10.7717/peerj.10629 (PMC7808263; doi:10.7717/peerj.10629)
Supplement: Supplemental Information 2 [file peerj-09-10629-s002.docx]

**Supplementary results**

In this section, we report the results when participants in the limit between two quartiles were allocated to the upper quartile. In this case, the range of scores were 32-49, for the first quartile; 50-55, for the second quartile; 56-62, for the third quartile; and 63-72, for the fourth quartile. Observers’ scores in the CFMT-Chinese were negatively associated with their scores in the PI-20 for the first [r = -0.25, *p = .05*, CI = -0.48 – 0.02] and fourth [r = -0.33, *p < .01*, CI = -0.53 – -0.10] quartiles. In contrast, the associations between the CFMT-Chinese and the PI-20 were not reliable either for the second or third quartile [Q2: r = 0.01, *p = .96*, CI = -0.24 – 0.25, Q3: r = 0.03, *p = .80*, CI = -0.20 – 0.26], or when both quartiles were combined [r = -.07, *p = .42*, CI = -0.23 – 0.10]. The reanalysis of Grey and colleagues’ results also showed reliable negative associations between the CFMT-Chinese and the PI-20 for the first [r = -0.26, *p < .01*, CI = -0.43 – -0.07] and fourth [r = -0.26., *p < .01*, CI = -0.42 – -0.08] quartiles. However, these associations were not reliable either for the second or third quartiles [Q2: r = -0.01, *p = .88*, CI = -0.20 – 0.17, Q3: r = 0.10, *p = .30*, CI = -0.09 – 0.29] or when both quartiles were combined [r = -0.09., *p = .18*, CI = -0.22 – 0.04].
