# Supplementary material for: Associations between self-reported and objective face recognition abilities are only evident in above- and below-average recognisers
Source: PeerJ. 2021 Jan 11;9:e10629. doi: 10.7717/peerj.10629 (PMC7808263; doi:10.7717/peerj.10629)
Supplement: Supplemental Information 3 [file peerj-09-10629-s003.docx]

**20题 - 脸盲症指数问卷 (PI20)**

以下道题查询关于您的脸部识别能力。您对以下的各项条目可能赞同或不赞同。请用1-5分的选项来评价这些条目并填写在横线上。请仔细阅读下列问题，然后诚实回答。谢谢。

| 1 | 2 | 3 | 4 | 5 |
| --- | --- | --- | --- | --- |
| 非常不同意 | 不同意 | 不确定 | 同意 | 非常同意 |

1. 我的脸部辨认能力比大部分人差。 ____

2. 我总是对于脸部有很糟糕的记忆力。 ____

3.当他人有明显的脸部特质，我比较容易进行脸部辨认。 ____

4.对于陌生人，即使之前有见过他，我经常认错。 ____

5.过去在学校，我需要很努力才能认出同学。 ____

6.当别人改变发型或是戴上帽子，我要认出他会有困难。 ____

7.有时候，我必须告知初次见面的人，我的脸部辨识能力不高。 ____

8.对我来说，在我脑海中描绘一个人的脸孔，是一件简单的事。 ____

9.当我看到一个人的脸时，我比大部分的人更能叫出他的名字。 ____

10.当我没有听到某人的声音时，我很难去认出他来。 ____

11.对于脸孔辨识的焦虑，让我不想出席特定的社交或某些正式场合。 ____

12.我比一般人需要花更多精力去记住脸孔。 ____

13.我很有信心从照片中认出我自己。 ____

14.我有时候很难跟上电影情节，因为我无法记住角色的外貌。 ____

15.我的朋友和家人认为我有很糟糕的脸部辨识能力。 ____

16.我经常因为认不出别人的样子而冒犯别人。 ____

17.即使有一群人在特定场合穿着相似的服装，我也能分辨出来。 ____

18.在家庭聚会，我经常搞不清楚谁是谁。 ____

19.我可以轻易地从名人的旧照片中认出他们，即使他们从前的样子和现在差异很大。 ____

20.就算是我熟悉的人，在不同情境下，我还是很难认出他来。 ____
